# Cross-species transmission of *Cryptosporidium* in wild rodents from the southern region of Zhejiang Province of China and its possible impact on public health[Fn FN1]

**DOI:** 10.1051/parasite/2024033

**Published:** 2024-07-01

**Authors:** Yanyan Jiang, Aiying Jiang, Guangxu Ren, Long Wang, Xianming Xin, Zhongying Yuan, Jiani Liu, Zhen Li, Yanbin Sun, Shanshan Zhou, Gang Lu, Huicong Huang, Wei Zhao

**Affiliations:** 1 National Institute of Parasitic Diseases, Chinese Center for Disease Control and Prevention (Chinese Center for Tropical Diseases Research), National Key Laboratory of Intelligent Tracking and Forecasting for Infectious Diseases, NHC Key Laboratory of Parasite and Vector Biology, National Center for International Research on Tropical Diseases, WHO Collaborating Centre for Tropical Diseases 200025 Shanghai China; 2 Department of Parasitology, School of Basic Medical Sciences, Wenzhou Medical University Wenzhou Zhejiang 325035 China; 3 Department of Pathogenic Biology, Hainan Medical University Haikou Hainan China; 4 Hainan Medical University – The University of Hong Kong Joint Laboratory of Tropical Infectious Diseases, Hainan Medical University Haikou Hainan China; 5 Key Laboratory of Tropical Translational Medicine of Ministry of Education, Hainan Medical University Haikou 571199 China

**Keywords:** *Cryptosporidium*, Molecular detection, Wild rodents, Zoonotic, Public health, China

## Abstract

Wild rodents serve as reservoirs for *Cryptosporidium* and are overpopulated globally. However, genetic data regarding *Cryptosporidium* in these animals from China are limited. Here, we have determined the prevalence and genetic characteristics of *Cryptosporidium* among 370 wild rodents captured from three distinct locations in the southern region of Zhejiang Province, China. Fresh feces were collected from the rectum of each rodent, and DNA was extracted from them. The rodent species was identified by PCR amplifying the vertebrate *cytochrome b* gene. *Cryptosporidium* was detected by PCR amplification and amplicon sequencing the small subunit of ribosomal RNA gene. Positive samples of *C. viatorum* and *C. parvum* were further subtyped by analyzing the 60-kDa glycoprotein gene. A positive *Cryptosporidium* result was found in 7% (26/370) of samples, involving five rodent species: *Apodemus agrarius* (36), *Niviventer niviventer* (75), *Rattus losea* (18), *R. norvegicus* (155), and *R. tanezumi* (86). Their respective *Cryptosporidium* positive rates were 8.3%, 5.3%, 11.1%, 7.1%, and 7.0%. Sequence analysis confirmed the presence of three *Cryptosporidium* species: *C. parvum* (4), *C. viatorum* (1), and *C. muris* (1), and two genotypes: *Cryptosporidium* rat genotype IV (16) and *C. mortiferum*-like (4). Additionally, two subtypes of *C. parvum* (IIdA15G1 and IIpA19) and one subtype of *C. viatorum* (XVdA3) were detected. These results demonstrate that various wild rodent species in Zhejiang were concurrently infected with rodent-adapted and zoonotic species/genotypes of *Cryptosporidium*, indicating that these rodents can play a role in maintaining and dispersing this parasite into the environment and other hosts, including humans.

## Abstract (fr)

Les rongeurs sauvages servent de réservoirs à *Cryptosporidium* et ont des grandes populations à l’échelle mondiale. Cependant, les données génétiques concernant *Cryptosporidium* chez ces animaux en Chine sont limitées. Ici, nous avons déterminé la prévalence et les caractéristiques génétiques de *Cryptosporidium* parmi 370 rongeurs sauvages capturés dans trois endroits distincts de la région sud de la province du Zhejiang, en Chine. Des excréments frais ont été collectés dans le rectum de chaque rongeur et l’ADN en a été extrait. L’espèce de rongeur a été identifiée par amplification par PCR du gène du cytochrome b des vertébrés. *Cryptosporidium* a été détecté par amplification PCR et séquençage d’amplicons de la petite sous-unité du gène de l’ARN ribosomal. Les échantillons positifs de *C. viatorum* et *C. parvum* ont ensuite été sous-typés en analysant le gène de la glycoprotéine de 60 kDa. Un résultat positif pour *Cryptosporidium* a été trouvé dans 7 % (26/370) des échantillons, impliquant cinq espèces de rongeurs : *Apodemus agrarius* (36), *Niviventer niviventer* (75), *Rattus losea* (18), *R. norvegicus* (155) et *R. tanezumi* (86). Leurs taux respectifs de positivité pour *Cryptosporidium* étaient de 8,3 %, 5,3 %, 11,1 %, 7,1 % et 7,0 %. L’analyse des séquences a confirmé la présence de trois espèces de *Cryptosporidium* : *C. parvum* (4), *C. viatorum* (1) et *C. muris* (1), et de deux génotypes : *Cryptosporidium* génotype IV de rat (16) et *C. mortiferum*-like (4). De plus, deux sous-types de *C. parvum* (IIdA15G1 et IIpA19) et un sous-type de *C. viatorum* (XVdA3) ont été détectés. Ces résultats démontrent que diverses espèces de rongeurs sauvages du Zhejiang sont simultanément infectées par des espèces/génotypes de *Cryptosporidium* zoonotiques et adaptés aux rongeurs, ce qui indique que ces rongeurs peuvent jouer un rôle dans le maintien et la dispersion de ce parasite dans l’environnement et d’autres hôtes, y compris les humains.

## Introduction

*Cryptosporidium*, a protozoan parasite that colonizes the intestines, is a significant contributor to moderate to chronic diarrhea and related fatalities among children under two years of age and immunocompromized patients (HIV-positive) [4, 34]. Additionally, *Cryptosporidium* has been identified as able to infect over 260 species of animals [24]. Humans can acquire this parasite through various routes, encompassing direct contact with infected individuals or animals, and ingestion of contaminated water and food [35]. The public health importance of cryptosporidiosis became evident with the global recognition of *Cryptosporidium* as the predominant waterborne parasite [8]. To effectively minimize the frequency of *Cryptosporidium* outbreaks, it is imperative to identify potential sources of infection and likely modes of transmission [13]. Thus, monitoring *Cryptosporidium* in different hosts becomes critical, especially in animal hosts in close contact with humans.

A wide range of molecular epidemiological strategies have been used to characterize *Cryptosporidium* species at species/genotype and subtype levels [24]. Currently, this parasite has been identified with an estimated 120 genotypes and 50 valid species [23, 26]. Moreover, almost 21 distinct species/genotypes of this parasite have been found in humans, primarily as a result of zoonotic transmission, where the infection is transmitted from animals to humans [23]. Rodents, as a key reservoir of *Cryptosporidium*, have attracted widespread attention, particularly wild ones, considering their involvement alongside various animals (domestic, stray, and wild) and water sources in maintaining the stability and continuity of the *Cryptosporidium* transmission cycle. The presented data indicate that rodents harbor a minimum of 25 species and 48 genotypes of *Cryptosporidium*. Among these, wild rodents harbor 21 species and 32 genotypes, with *C. parvum* being the most common species [36]. Therefore, wild rodents potentially play a pivotal role in the transmission of zoonotic *Cryptosporidium* species. Despite this understanding, significant gaps exist concerning the incidence of *Cryptosporidium* infection in various nations and territories. For instance, in China, molecular studies on *Cryptosporidium* species in wild rodent species have been restricted to a small number of species [15, 22, 36].

*Cryptosporidium* species have been observed to exhibit a high prevalence in diverse animal species, including pigs, cattle, chickens, and horses, within the geographical region of Zhejiang Province, China [9, 28, 33, 40]. Moreover, they have also been found in patients with diarrhea, as well as in the source water of several cities of this province [2, 21, 37]. However, currently, there is only one study that confirms the presence of this parasite in *R. norvegicus* from Jiaxing City in Zhejiang [22]. The objective of the present study was to investigate the distribution, prevalence, and genetic characteristics of *Cryptosporidium* species among wild rodents residing in southern Zhejiang Province.

## Materials and methods

### Ethics

The present study was conducted as per the recommendations of the Chinese Laboratory Animal Administration Act (1988), which regulates the ethical handling and use of animals in scientific studies. All protocols were carefully examined and approved by the Research Ethics Committee of Wenzhou Medical University (SCILLSC-2021-01).

### Sample collection

**Figure 1:**
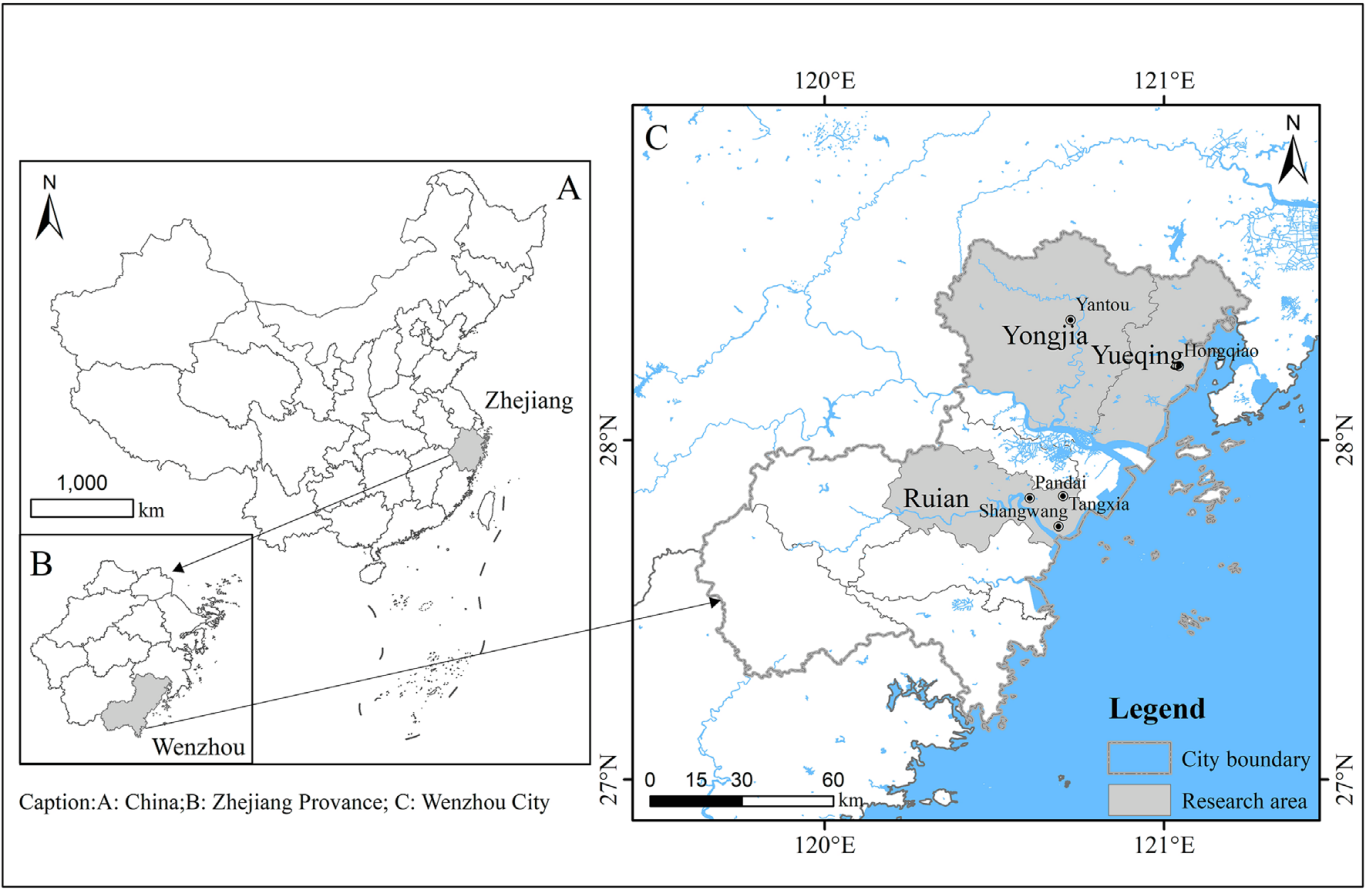
Map of rodent sampling locations in Wenzhou, Zhejiang Province, China. The figure was originally designed by the authors under ArcGIS 10.4 software. The original vector diagram imported in ArcGIS was adapted from the National Geomatics Center of China (http://www.ngcc.cn). The map has been modified and assembled according to permission and attribution guidelines.

A total of 370 wild rodents were trapped in three distinct locations within rural areas immediately adjacent to human habitations in Zhejiang Province, between April 1 and October 14, 2023 (specifically, encompassing the second week of April, June, August, and October in the year 2023) (Fig. 1). Among these, 68 were caught in Yueqing (Hongqiao), 102 in Yongjia (Yantou), and 200 in Rui’an (Tangxia, Pandai, Shangwang). All wild rodents were trapped in cage traps baited with deep-fried dough sticks. In each designated location, around 50 cage traps were deployed at sunset and collected before sunrise. The traps were positioned in a linear setup, with 5 m between each trap, forming transects. All rodents were shifted to the controlled laboratory environment within 48 h following their capture and euthanized *via* CO_2_ inhalation. Data related to the collection time and region was noted after these rodents were captured *via* trapping. Following that, a fresh feces sample (500 mg) was obtained immediately from the rectum of each rodent. The sample was then stored in ice boxes and shifted to the laboratory, where its DNA was extracted within a week.

### DNA extraction

As per the manufacturer’s recommendations, genomic DNA was isolated from each processed sample (200 mg) *via* a QIAamp DNA Mini Stool Kit (QIAGEN, Hilden, Germany). To achieve a significant yield of DNA, the lysate temperature was elevated to 95 °C. Before the PCR analysis, the DNA reconstituted in 200 μL of AE elution buffer (supplied with the kit) was kept at −20 °C.

### Identification of rodent species

The rodent species were identified *via* PCR amplification of the vertebrate *cytochrome b* (*cytb*) gene with 421 bp amplified from fecal DNA. The primer design and PCR conditions were in line with the guidelines defined by Verma and Singh (2003) [27]. Each PCR reaction was comprised of 35 cycles, which included denaturation at 94 °C for 30 s, annealing at 51 °C for 30 s, and extension at 72 °C for 30 s. An initial denaturation step was also performed at 94 °C for 5 min, followed by the completion of a final extension at 72 °C for 5 min.

### *Cryptosporidium* genotyping and subtyping

Nested PCR was performed on all isolated DNA with a specific target, using an 830 bp fragment of the partial small subunit of ribosomal RNA (*SSU rRNA*) gene of *Cryptosporidium* for amplification. Based on a previous description, primers were synthesized [32]. The 60-kDa glycoprotein (*gp60*) gene was amplified using nested PCR, enabling the further subtyping of positive isolates of *C. parvum* and *C. viatorum* using the same primers previously designed by Alves et al. (2003) and Stensvold et al. (2015), respectively [1, 25]. In every PCR amplification process, TaKaRa Taq DNA Polymerase (TaKaRa Bio Inc., Tokyo, Japan) was utilized. To ensure the validity of the reactions, positive controls, which contain *C. bailey* DNA derived from chickens, and negative controls, where no DNA template is included, were incorporated in every PCR reaction. Before sequencing, secondary PCR products were observed on 1.5% agarose gels, followed by staining with GelRed (Biotium, Fremont, CA, USA).

### Sequencing and phylogenetic analysis

The commercial sequencing of amplified products of *SSU rRNA* and *gp60* genes of *Cryptosporidium* spp. was performed by Sangon Biotech (Shanghai) Co., Ltd. (Shanghai, China). Two-way sequencing was used to validate the accuracy of the sequence. To examine the species and subtype of *Cryptosporidium* species, the identified sequences were aligned with the reference sequences obtained from the National Center for Biotechnology (https://www.ncbi.nlm.nih.gov/) using ClustalX 2.0 (http://www.clustal.org/). In MEGA 11, a neighbor-joining (NJ) method with a Kimura 2-parameter model was used to conduct phylogenetic analyses, with the objective of assessing the phylogenetic relationships among the sequences obtained in this study and pertinent reference sequences available in GenBank. The clusters’ stability was evaluated using 1000 replicates and Bootstrap analysis.

### Statistical analyses

Data analysis was performed with SPSS version 22.0 (SPSS Inc., Chicago, IL, USA). The chi-square test was utilized to compare the prevalence of *Cryptosporidium* spp. between areas, gender, rodent species and season groups, respectively. A *p*-value < 0.05 was considered indicative of statistical significance.

### Nucleotide sequence accession numbers

The nucleotide sequences of *Cryptosporidium* obtained in this study were deposited in the GenBank database under accession numbers PP038021 to PP038023 and PP038026 to PP038028 for *SSU rRNA*, and PP104938 to PP104940 for *gp60*.

## Results

### Study population

This study used PCR and sequencing analysis of the *cytb* gene to identify five species of rodents, including *Rattus norvegicus* (*n* = 155), *R. tanezumi* (*n* = 86), *Niviventer niviventer* (*n* = 75), *Apodemus agrarius* (*n* = 36) and *R. losea* (*n* = 18) (Table 1 and Table S1). The majority of samples were obtained in the summer (43.2%, 160/370), then in spring (32.2%, 119/370) and autumn (24.6%, 91/370); none were collected in the winter. The sex of the rodents was reported as 52.7% (195/370) females and 47.3% (175/370) males (Table 1 and Table S1).

**Table 1:** Prevalence and species/genotype of *Cryptosporidium* in the investigated rodent by species, season, gender, and location.

Category	Positive/examined (%)	*Cryptosporidium* spp./genotype (n)
Rodent species		
*Apodemus agrarius*	3/36 (8.3)	*C. parvum* (2), *C. muris* (1)
*Niviventer niviventer*	4/75 (5.3)	*Cryptosporidium* rat genotype IV (4)
*Rattus losea*	2/18 (11.1)	*Cryptosporidium* rat genotype IV (2)
*Rattus norvegicus*	11/155 (7.1)	*C. mortiferum*-like (4), *Cryptosporidium* rat genotype IV (4), *C. parvum* (2), *C. viatorum* (1)
*Rattus tanezumi*	6/86 (7.0)	*Cryptosporidium* rat genotype IV (6)
Season		
Spring	11/119 (9.2)	*Cryptosporidium* rat genotype IV (4), *C. mortiferum*-like (4), *C. parvum* (2), *C. viatorum* (1)
Summer	8/160 (5.0)	*Cryptosporidium* rat genotype IV (5), *C. parvum* (2), *C. muris* (1)
Autumn	7/91 (7.7)	*Cryptosporidium* rat genotype IV (7)
Gender		
Female	9/195 (4.6)	*Cryptosporidium* rat genotype IV (3), *C. mortiferum*-like (4), *C. parvum* (1), *C. muris* (1)
Male	17/175 (9.7)	*Cryptosporidium* rat genotype IV (13), *C. parvum* (3), *C. viatorum* (1)
Location		
Yueqing	10/68 (14.7)	*Cryptosporidium* rat genotype IV (7), *C. parvum* (2), *C. viatorum* (1)
Yongjia	3/102 (2.9)	*Cryptosporidium* rat genotype IV (2), *C. mortiferum*-like (1)
Rui’an	13/200 (6.5)	*Cryptosporidium* rat genotype IV (7), *C. mortiferum*-like (3), *C. parvum* (2), *C. muris* (1)
Total	26/370 (7.0)	*Cryptosporidium* rat genotype IV (16), *C. parvum* (4), *C. mortiferum*-like (4), *C. viatorum* (1), *C. muris* (1)

### Prevalence of *Cryptosporidium* infection

Nested PCR was conducted on 370 fecal samples to evaluate the existence of *Cryptosporidium* species *via* the *SSU rRNA* gene. A total of 26 samples tested positive for this parasite with an average (7.0%) infection rate. *Cryptosporidium* existed in all three areas, with infection rates of 14.7% (Yeqing), 2.9% (Yongjia), and 6.5% (Rui’an) (Table 1 and Table S1). Statistical analysis revealed significant variations in the prevalence of *Cryptosporidium* among the three regions (*χ*^2^ = 8.829; df = 2; *p* = 0.012).

The infection rates of *Cryptosporidium* vary among rodent species, ranging from 5.3% (4/75) in *N. niviventer* to 11.1% (2/18) in *R. losea*, with 7.1% (11/155) in *R. norvegicus*, 7.0% (6/86) in *R. tanezumi*, and 8.3% (3/36) in *A. agrarius* (Table 1 and Table S1). The highest detection rate of *Cryptosporidium* in rodents collected in spring, reaching 9.2% (11/119), followed by 7.7% (7/91) in autumn, and 5.5% (8/160) in summer (Table 1 and Table S1). However, the difference between infection rates of *Cryptosporidium* in the groups of rodent species and seasons was not regarded as statistically significant (*p* > 0.05). In relation to gender, the incidence of *Cryptosporidium* was comparatively lower in female (4.6%) (9/195) than in male (9.7%; 17/175) rodents, but without statistical significance (*χ*^2^ = 3.67; df = 1; *p* = 0.06) (Table 1 and Table S1).

### *Cryptosporidium* species/genotypes distribution

Five *Cryptosporidium* species or genotypes were detected, including *Cryptosporidium* rat genotype IV (*n* = 16), *C. parvum* (*n* = 4), *C. mortiferum*-like genotype (*n* = 4), *C. viatorum* (*n* = 1), and *C. muris* (*n* = 1) (Table 1 and Table S1). The prevalence of *Cryptosporidium* rat genotype IV was observed to be predominant (61.5%; 16/26) among the wild rodent population. This genotype was detected in four out of the five rodent species, except *A. agrarius* (Table 1 and Table S1). Of the four *C. parvum* isolates, two each were found in *R. norvegicus* and *A. agrarius. Cryptosporidium mortiferum*-like was only found in *R. norvegicus*, while *C. viatorum* and *C. muris* were identified in a single *R. norvegicus* and *A. agrarius*, respectively (Table 1 and Table S1).

The sampling sites exhibit differences in the distribution of *Cryptosporidium* species. Specifically, Yeqing yielded the *Cryptosporidium* rat genotype IV, *C. viatorum* and *C. parvum*. Yongjia yielded *C. mortiferum*-like and *Cryptosporidium* rat genotype IV. While *Cryptosporidium* rat genotype IV, *C. parvum*, *C. muris* and *C. mortiferum*-like were discovered in Rui’an (Table 1 and Table S1).

Meanwhile, *Cryptosporidium* rat genotype IV was detected throughout all three seasons, whereas *C. muris* was exclusively detected in the summer. Conversely, *C. viatorum* and *C. mortiferum-like* were only detected in the spring (Table 1). In terms of gender, both male and female rodents were found to harbor *Cryptosporidium* rat genotype IV and *C. parvum*, whereas only female rodents were found to possess *C. muris* and *C. mortiferum*-like, and male rodents were found to harbor *C. viatorum* (Table 1 and Table S1).

### Genetic identification of *Cryptosporidium* species/genotypes

**Figure 2:**
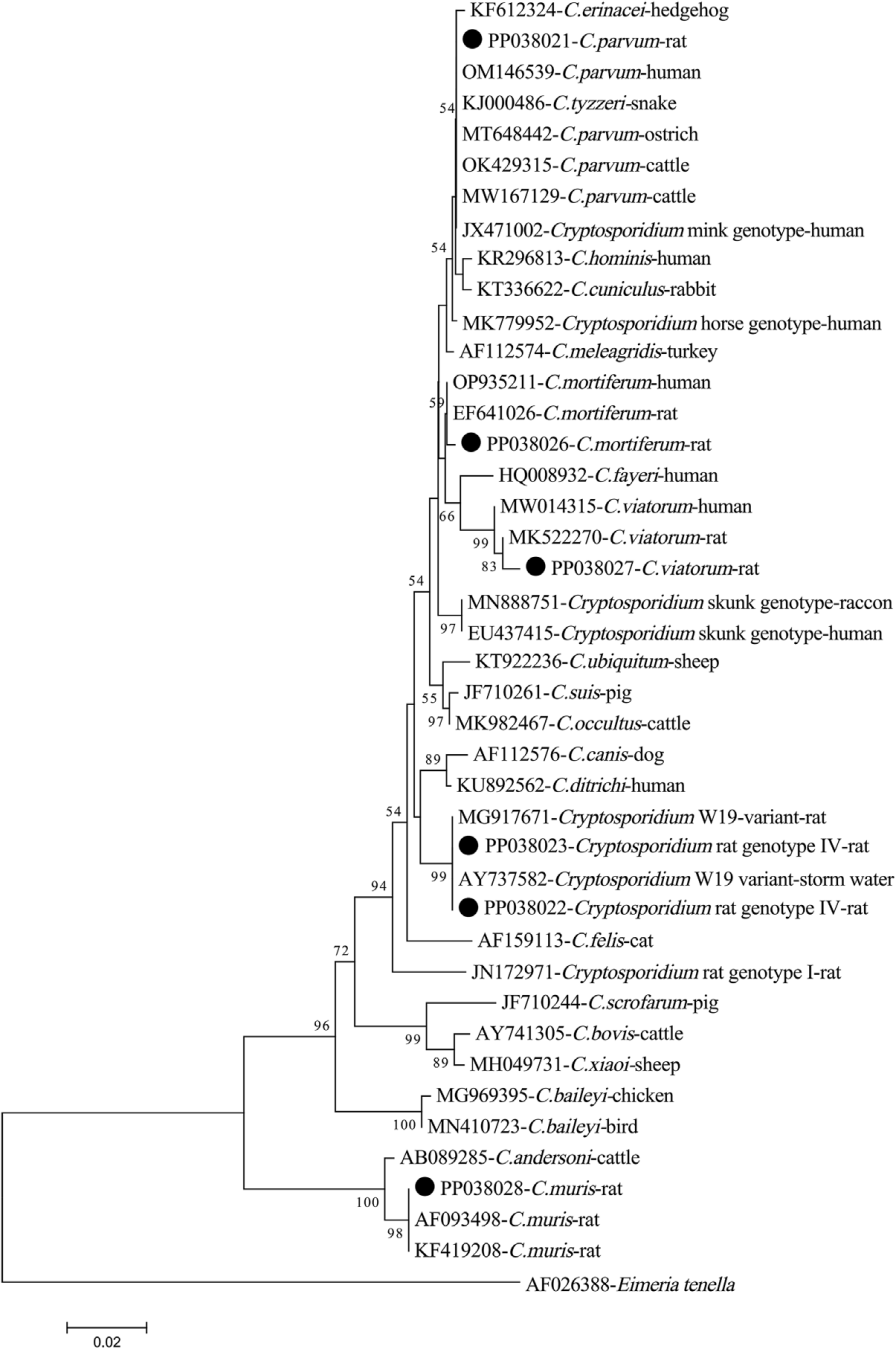
Phylogenetic tree of *Cryptosporidium* species based on SSU rRNA sequences. The tree was generated using a neighbor-joining analysis, with genetic distances calculated *via* the Kimura 2-parameter model. Bootstrap values (>50%) derived from 1000 replicates are displayed to the left of the nodes for reliability assessment. The sequences generated in the present study are indicated with the solid circles.

At the *SSU rRNA* locus, among the 16 sequences of *Cryptosporidium* rat genotype IV, 10 and six sequences were identical and had 100% similarity with the sequence AY737582 of genotype W19 variant in storm water from the USA and MG917671 of genotype W19 variant in brown rats from China, respectively (Table S2). Four sequences of *C. parvum* were identical and had 100% similarity with the sequence OM146539 of *C. parvum* in humans from Sweden, as well as eight other sequences in *Macaca mulatta* or bamboo rats from China (Table S2). The four *C. mortiferum*-like isolates possessed identical sequences which have not been documented previously and exhibit a sequence similarity of 98.78% to the *C. mortiferum* sequence (OP935211) detected in humans from the USA (Table S2). The sequence of *C. viatorum* was similar and it has not been documented previously and exhibited homology of 99.61% to the sequence MK522270 of *C. viatorum* isolated in *Berylmys bowersi* from China (Table S2). The sequence of *C. muris* exhibited 100% identity to KF419208, which is found in *R. norvegicus* from China (Table S2). In the phylogenetic tree, the sequences belonging to the same species were shown to form distinct clusters, as depicted in Figure 2.

**Figure 3:**
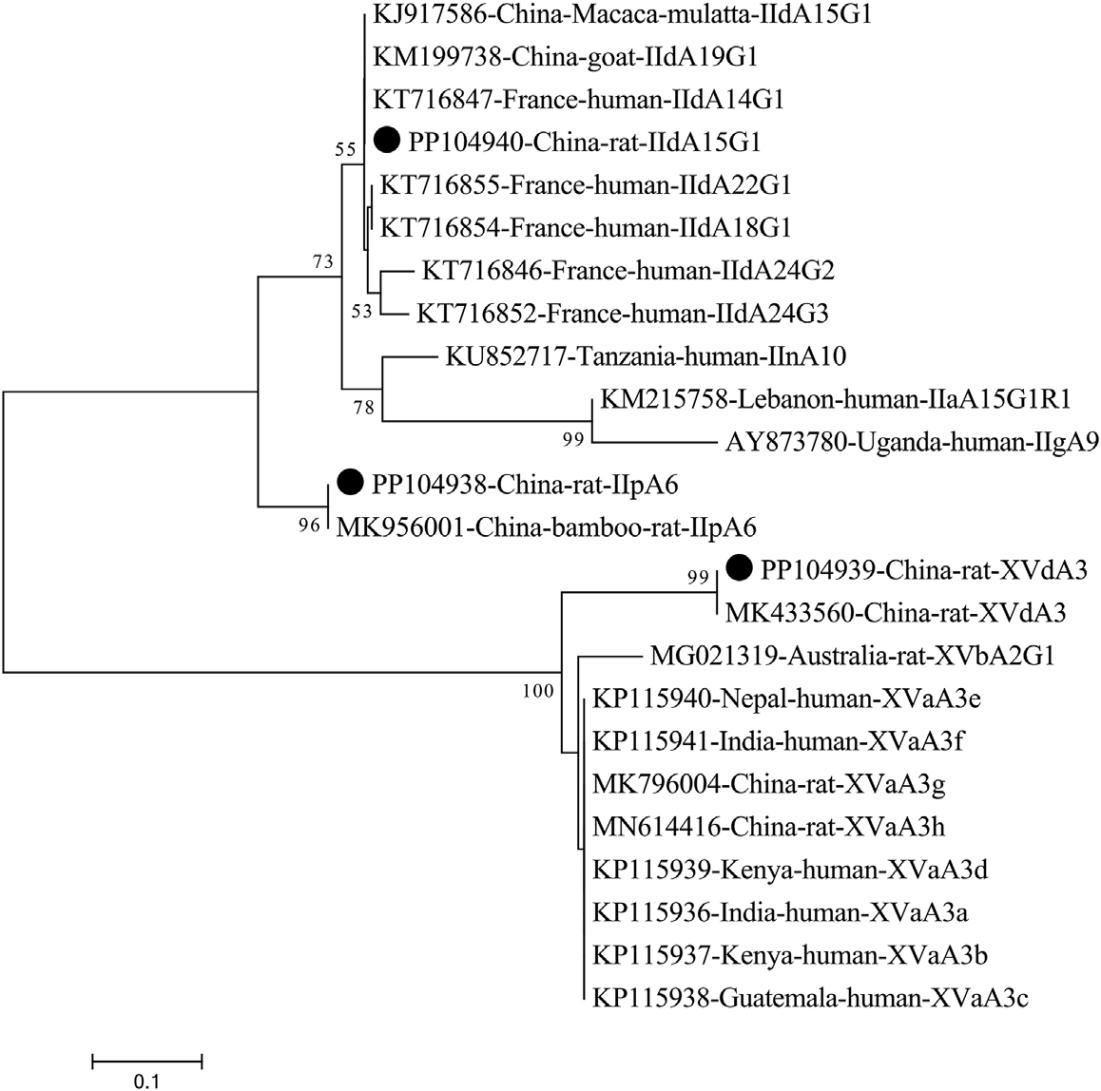
Phylogenetic relationships of gp60 subtypes of *C. parvum* and *C. viatorum* identified in the study and other gp60 subtype sequences deposited in GenBank, as inferred by a neighbor-joining analysis of gp60 sequences based on the genetic distance by the Kimura 2-parameter model. The numbers displayed on the branches represent the percentage bootstrapping outcomes derived from 1000 replicates. The sequences generated in the present study are indicated with solid circles.

At the *gp60* gene locus, successful amplification was achieved for *C. viatorum* and three *C. parvum*-positive isolates. Two of the three gp60 sequences obtained from *C. parvum* exhibited 100% resemblance to the sequence of *M. mulatta* in China, from which *C. parvum* subtype IIdA15G1 (KJ917586) was identified (Table S2). The other one, gp60 sequence of *C. parvum,* shared 100% similarity to subtype IIpA6 (MK956001) from bamboo rats in China (Table S2). The *C. viatorum* sample had the same gp60 sequence which has not been documented in the previous literature and possesses a nucleotide similarity of 99.51% with the well-documented subtype XVdA3 (MK433560) of *C. viatorum* originating from *Leopoldamys edwardsi* in China (Table S2). Figure 3 presents the phylogenetic tree, showing the genetic correlations among the gp60 subtypes of *C. parvum* and *C. viatorum*.

## Discussion

In the present study, the average rate of *Cryptosporidium* infection among the identified rodents was 7.0%, which was found to be lower than the aggregated global rate for wild rodents (20.5%), as determined by Zhang et al. [36]. In China, *Cryptosporidium* has been found in a variety of rodents, where the infection rates vary by their types, such as 4.0–73.9% in wild rodents, 2.1–29.5% in farmed rodents, 0.6–8.6% in lab rodents and 1.4–100% in pet rodents [22, 36]. The differences in rodent species, detection strategies, animal age, sample size, and study locations might be responsible for the disparity in prevalence.

The present study identified five *Cryptosporidium* species/genotypes including *Cryptosporidium* rat genotype IV, *C. mortiferum*-like, *C. parvum*, *C. viatorum* and *C. muris*. Multiple studies have demonstrated that rats serve as a predominant host species for *Cryptosporidium* rat genotype IV (formerly known as *Cryptosporidium* environmental sequence, *Cryptosporidium* genotypes W19, or W19 variant) [36, 39]. *Cryptosporidium* rat genotype IV has previously been found in Asian house rats, Edward’s long-tailed rats, Muridae, and brown rats from China [6, 38], and it has also been identified in rats from Japan, Spain and Sweden [3, 14, 17]. However, despite the discovery of *Cryptosporidium* rat genotype IV in Asiatic black bears and cats from China and in one-humped camels from Egypt, limited data exist regarding the chances for infection of humans and other animals by *Cryptosporidium* rat genotype IV [7, 19, 29]. Consequently, the possibility of this genotype inducing disease in livestock or humans remains uncertain. Further systemic molecular epidemiological studies into *Cryptosporidium* species with a wider range of hosts are required in the future to identify the exact host distribution of *Cryptosporidium* rat genotype IV.

Zoonotic species include *C. muris*, *C. viatorum* and *C. parvum*, due to the extensive documentation of their infections in humans and a diverse array of mammalian hosts [23]. For example, *C. parvum*, which is prevalent in rodents worldwide, has been consistently identified in wildlife, having infected over 40 species of wild animals [23, 36]. In China 16.7% of human cases (44/263) of cryptosporidiosis were attributed to *C. parvum*, a prevalent pathogen in farmed animals, including cattle, sheep, and goats [12, 20]. Further, 18.7% of rodent-derived *Cryptosporidium* cases (189/1010) had been confirmed to be caused by *C. parvum* [36]. Initially, *C. viatorum* was detected in travelers from the Indian subcontinent who had arrived in the United Kingdom [7]. More than 13 countries, including China, have reported cases of *C. viatorum* in humans [25, 31]. Further analysis revealed the presence of *C. viatorum* in several rodent species, including *R. rattus* from France, *R. lutreolus* from Australia and *Leopoldamys edwardsi* and *Berylmys bowersi* from China [10, 16, 38]. Additionally, *C. muris* has been extensively documented in various mammalian hosts, such as rodents, felids, canids, equids, suids, non-human primates, etc. [23]. The transmission of *C. parvum*, *C. viatorum* and *C. muris* from wild rodent species to humans and other animals *via* cross-species contact could, therefore, not be ignored.

This study identified a novel genotype in *R. norvegicus* that shares genetic similarities to *C. mortiferum* (*Cryptosporidium* chipmunk genotype I), named *C. mortiferum-*like. The sequences of *C. mortiferum*-like discovered in this study have not been previously reported in the literature. However, it is well known that *C. mortiferum* can infect people, and several human cases have been reported [11, 23, 26]. Therefore, *C. mortiferum*-like is highly likely to also have the ability to infect people, and of course, clear evidence needs to be provided through more research in the future. The discovery of novel sequences of *Cryptosporidium* in *R. norvegicus* suggests the existence of some novel *Cryptosporidium* species/genotypes in wild rodents. This is primarily due to the order of rodents having the most diverse of all mammalian groups.

In the evaluation of *C. parvum* in both animals and humans, subtyping tools are frequently used. The transmission of *C. parvum* between animals and humans was enhanced *via* the application of subtype-specific molecular diagnostic tools [12, 24]. At least 15 subtype families for *C. parvum* were detected *via gp60* gene analysis, including IIa-IIi and IIk-IIp [12]. In China, at least 20 subtypes have been identified, with IIaA15G2R1, IIaA15G2R2, IIaA13G2R2, IIdA15G1 and IIdA14G1 being found in humans [12, 20]. Wild rodents were examined in the present study that observed two subtypes (IIdA15G1 and IIpA6) of *C. parvum*. The subtype IIdA15G1 is one of the prevalent subtypes found in cattle, exhibiting a diverse geographic distribution across China [12]. Its elevated mortality rate among pre-weaned dairy calves in China has been attributed to multiple outbreaks of cryptosporidiosis [5, 12]. Furthermore, the subtype was subsequently detected in non-human primates and humans in China, which provides further evidence for the possibility of zoonotic transmission [12, 30, 37]. Thus, IIdA15G1-infected wild rodents pose a potential risk to both humans and other animals. However, IIpA6 has only been detected in bamboo rodents thus far, and its potential to infect humans and livestock is unknown [18]. A comprehensive understanding of the host range of subtype IIpA6 of *Cryptosporidium* species would require systematic molecular epidemiological studies across a wider range of hosts.

## Conclusions

The present study provided evidence of the presence of *Cryptosporidium* in five species of wild rodents in Zhejiang, China, with an average infection rate of 7.0%. The presented molecular findings suggest that *Cryptosporidium* rat genotype IV predominantly infect wild rodents. As a result, these rodents have a restricted capacity to serve as natural reservoirs for human infections. In contrast, the discovery of *C. muris*, *C. parvum*, *C. mortiferum*-like, *C. viatorum* and *C. viatorum* suggests a connection between rodents and humans. This finding demonstrates that animals infected with *Cryptosporidium* have substantial zoonotic potential and indicates that wild rodents could serve as a reservoir for human cryptosporidiosis caused by the *Cryptosporidium* species above.

## Supplementary Tables

The supplementary Tables S1 and S2 for this article can be found at https://www.parasite-journal.org/10.1051/parasite/2024033/olm. *Table S1*: Prevalence and species of *Cryptosporidium* in the investigated wild rodents by species, season, gender, and location. *Table S2*: Similarity analysis of SSUrRNA and Gp60 sequences of *Cryptosporidium* obtained in this study.

## References

[1] Alves M, Xiao L, Sulaiman I, Lal AA, Matos O, Antunes F. 2003. Subgenotype analysis of *Cryptosporidium* isolates from humans, cattle, and zoo ruminants in Portugal. Journal of Clinical Microbiology, 41(6), 2744–2747. 12791920 10.1128/JCM.41.6.2744-2747.2003 PMC156540

[2] An W, Zhang D, Xiao S, Yu J, Yang M. 2011. Quantitative health risk assessment of *Cryptosporidium* in rivers of southern China based on continuous monitoring. Environmental Science & Technology, 45(11), 4951–4958. 21557575 10.1021/es103981w

[3] Backhans A, Jacobson M, Hansson I, Lebbad M, Lambertz ST, Gammelgård E, Saager M, Akande O, Fellström C. 2013. Occurrence of pathogens in wild rodents caught on Swedish pig and chicken farms. Epidemiology and Infection, 141(9), 1885–1891. 23174339 10.1017/S0950268812002609 PMC9151424

[4] Bouzid M, Hunter PR, Chalmers RM, Tyler KM. 2013. *Cryptosporidium* pathogenicity and virulence. Clinical Microbiology Reviews, 26(1), 115–134. 23297262 10.1128/CMR.00076-12 PMC3553671

[5] Cui Z, Wang R, Huang J, Wang H, Zhao J, Luo N, Li J, Zhang Z, Zhang L. 2014. Cryptosporidiosis caused by *Cryptosporidium parvum* subtype IIdA15G1 at a dairy farm in Northwestern China. Parasites & Vectors, 7, 529. 25430474 10.1186/s13071-014-0529-z PMC4254006

[6] Deng L, Chai Y, Luo R, Yang L, Yao J, Zhong Z, Wang W, Xiang L, Fu H, Liu H, Zhou Z, Yue C, Chen W, Peng G. 2020. Occurrence and genetic characteristics of *Cryptosporidium* spp. and *Enterocytozoon bieneusi* in pet red squirrels (*Sciurus vulgaris*) in China. Scientific Reports, 10(1), 1026. 31974403 10.1038/s41598-020-57896-w PMC6978461

[7] Elwin K, Hadfield SJ, Robinson G, Crouch ND, Chalmers RM. 2012. *Cryptosporidium viatorum* n. sp. (*Apicomplexa: Cryptosporidiidae*) among travellers returning to Great Britain from the Indian subcontinent, 2007–2011. International Journal for Parasitology, 42(7), 675–682. 22633952 10.1016/j.ijpara.2012.04.016

[8] Fayer R. 2004. *Cryptosporidium*: A water-borne zoonotic parasite. Veterinary Parasitology, 126(1–2), 37–56. 15567578 10.1016/j.vetpar.2004.09.004

[9] Feng X, Deng J, Zhang Z, Yu F, Zhang J, Shi T, Sun H, Qi M, Liu X. 2023. Dominant infection of *Cryptosporidium baileyi* in broiler chickens in Zhejiang Province, China. Parasitology Research, 122(9), 1993–2000. 37347286 10.1007/s00436-023-07898-0

[10] García-Livia K, Fernández-Álvarez Á, Feliu C, Miquel J, Quilichini Y, Foronda P. 2022. *Cryptosporidium* spp. in wild murids (*Rodentia*) from Corsica, France. Parasitology Research, 121(1), 345–354. 34816301 10.1007/s00436-021-07369-4 PMC8748365

[11] Guo Y, Cebelinski E, Matusevich C, Alderisio KA, Lebbad M, McEvoy J, Roellig DM, Yang C, Feng Y, Xiao L. 2015. Subtyping novel zoonotic pathogen *Cryptosporidium chipmunk* genotype I. Journal of Clinical Microbiology, 53(5), 1648–1654. 25762767 10.1128/JCM.03436-14 PMC4400750

[12] Guo Y, Ryan U, Feng Y, Xiao L. 2022. Emergence of zoonotic *Cryptosporidium parvum* in China. Trends in Parasitology, 38(4), 335–343. 34972653 10.1016/j.pt.2021.12.002

[13] Helmy YA, Hafez HM. 2022. Cryptosporidiosis: From prevention to treatment, a narrative review. Microorganisms, 10(12), 2456. 36557709 10.3390/microorganisms10122456 PMC9782356

[14] Hikosaka K, Nakai Y. 2005. A novel genotype of *Cryptosporidium muris* from large Japanese field mice, *Apodemus speciosus*. Parasitology Research, 97(5), 373–379. 16151744 10.1007/s00436-005-1459-7

[15] Hu B, Wang J, Zhang S, Wang B, Xing Y, Han S, He H. 2022. Novel genotypes of *Cryptosporidium* and *Enterocytozoon bieneusi* detected in plateau zokors (*Myospalax baileyi*) from the Tibetan Plateau. International Journal for Parasitology: Parasites and Wildlife, 19, 263–268. 36388721 10.1016/j.ijppaw.2022.11.002 PMC9661441

[16] Koehler AV, Wang T, Haydon SR, Gasser RB. 2018. *Cryptosporidium viatorum* from the native Australian swamp rat *Rattus lutreolus* – An emerging zoonotic pathogen?. International Journal for Parasitology: Parasites and Wildlife, 7(1), 18–26. 29556470 10.1016/j.ijppaw.2018.01.004 PMC5853523

[17] Köster PC, Dashti A, Bailo B, Muadica AS, Maloney JG, Santín M, Chicharro C, Migueláñez S, Nieto FJ, Cano-Terriza D, García-Bocanegra I, Guerra R, Ponce-Gordo F, Calero-Bernal R, González-Barrio D, Carmena D. 2021. Occurrence and genetic diversity of protist parasites in captive non-human primates, zookeepers, and free-living sympatric rats in the Córdoba zoo conservation centre, Southern Spain. Animals, 11(3), 700. 33807707 10.3390/ani11030700 PMC8035673

[18] Li F, Zhao W, Zhang C, Guo Y, Li N, Xiao L, Feng Y. 2020. *Cryptosporidium* species and *C. parvum* subtypes in farmed bamboo rats. Pathogens, 9(12), 1018. 33276616 10.3390/pathogens9121018 PMC7761605

[19] Li J, Dan X, Zhu K, Li N, Guo Y, Zheng Z, Feng Y, Xiao L. 2019. Genetic characterization of *Cryptosporidium* spp. and *Giardia duodenalis* in dogs and cats in Guangdong, China. Parasites & Vectors, 12(1), 571. 31783765 10.1186/s13071-019-3822-z PMC6884805

[20] Liu A, Gong B, Liu X, Shen Y, Wu Y, Zhang W, Cao J. 2020. A retrospective epidemiological analysis of human *Cryptosporidium* infection in China during the past three decades (1987–2018). PLoS Neglected Tropical Diseases, 14(3), e0008146. 32226011 10.1371/journal.pntd.0008146 PMC7145189

[21] Liu H, Ni H, Liu S, Shen Y, Wang R, Cao J, Yin J. 2023. First report on occurrence and genotypes of *Enterocytozoon bieneusi*, *Cryptosporidium* spp. and *Cyclospora cayetanensis* from diarrheal outpatients in Ningbo, Southeast China. Microbial Pathogenesis, 174, 105952. 36528327 10.1016/j.micpath.2022.105952

[22] Ni HB, Sun YZ, Qin SY, Wang YC, Zhao Q, Sun ZY, Zhang M, Yang D, Feng ZH, Guan ZH, Qiu HY, Wang HX, Xue NY, Sun HT. 2021. Molecular detection of *Cryptosporidium* spp. and *Enterocytozoon bieneusi* infection in wild rodents from six provinces in China. Frontiers in Cellular and Infection Microbiology, 11, 783508. 34900760 10.3389/fcimb.2021.783508 PMC8656357

[23] Ryan U, Zahedi A, Feng Y, Xiao L. 2021. An update on zoonotic *Cryptosporidium* species and genotypes in humans. Animals, 11(11), 3307. 34828043 10.3390/ani11113307 PMC8614385

[24] Ryan UM, Feng Y, Fayer R, Xiao L. 2021. Taxonomy and molecular epidemiology of *Cryptosporidium* and *Giardia* – A 50 year perspective (1971–2021). International Journal for Parasitology, 51(13–14), 1099–1119. 34715087 10.1016/j.ijpara.2021.08.007

[25] Stensvold CR, Elwin K, Winiecka-Krusnell J, Chalmers RM, Xiao L, Lebbad M. 2015. Development and application of a gp60-based typing assay for *Cryptosporidium viatorum*. Journal of Clinical Microbiology, 53(6), 1891–1897. 25832304 10.1128/JCM.00313-15 PMC4432048

[26] Tůmová L, Ježková J, Prediger J, Holubová N, Sak B, Konečný R, Květoňová D, Hlásková L, Rost M, McEvoy J, Xiao L, Santín M, Kváč M. 2023. *Cryptosporidium mortiferum* n. sp. (Apicomplexa: Cryptosporidiidae), the species causing lethal cryptosporidiosis in Eurasian red squirrels (*Sciurus vulgaris*). Parasites & Vectors, 16(1), 235. 37454101 10.1186/s13071-023-05844-8 PMC10349434

[27] Verma SK, Singh L. 2003. Novel universal primers establish identity of an enormous number of animal species for forensic application. Molecular Ecology Notes, 3, 28–31.

[28] Wang L, Xue X, Li J, Zhou Q, Yu Y, Du A. 2014. Cryptosporidiosis in broiler chickens in Zhejiang Province, China: Molecular characterization of oocysts detected in fecal samples. Parasite, 21, 36. 25075975 10.1051/parasite/2014035 PMC4115479

[29] Wang SN, Sun Y, Zhou HH, Lu G, Qi M, Liu WS, Zhao W. 2020. Prevalence and genotypic identification of *Cryptosporidium* spp. and *Enterocytozoon bieneusi* in captive Asiatic black bears (*Ursus thibetanus*) in Heilongjiang and Fujian provinces of China. BMC Veterinary Research, 16(1), 84. 32151253 10.1186/s12917-020-02292-9 PMC7063761

[30] Wang T, Wei Z, Zhang Y, Zhang Q, Zhang L, Yu F, Qi M, Zhao W. 2022. Molecular detection and genetic characterization of *Cryptosporidium* in kindergarten children in Southern Xinjiang, China. Infection Genetics and Evolution, 103, 105339. 10.1016/j.meegid.2022.105339 35840104

[31] Wu Y, Gong B, Liu X, Jiang Y, Cao J, Yao L, Li H, Liu A, Shen Y. 2020. Identification of uncommon *Cryptosporidium viatorum* (a novel subtype XVcA2G1c) and *Cryptosporidium andersoni* as well as common *Giardia duodenalis* assemblages A and B in Humans in Myanmar. Frontiers in Cellular and Infection Microbiology, 10, 614053. 33324584 10.3389/fcimb.2020.614053 PMC7724083

[32] Xiao L, Escalante L, Yang C, Sulaiman I, Escalante AA, Montali RJ, Fayer R, Lal AA. 1999. Phylogenetic analysis of *Cryptosporidium* parasites based on the small-subunit rRNA gene locus. Applied and Environmental Microbiology, 65(4), 1578–1583. 10103253 10.1128/aem.65.4.1578-1583.1999 PMC91223

[33] Xu C, Wei Z, Tan F, Liu A, Yu F, Zhao A, Zhang L, Qi M, Zhao W. 2023. Molecular detection and genetic characteristics of *Cryptosporidium* spp. in Chinese racehorses. Equine Veterinary Journal, 55(3), 474–480. 35680650 10.1111/evj.13605

[34] Yang X, Guo Y, Xiao L, Feng Y. 2021. Molecular epidemiology of human cryptosporidiosis in low- and middle-income countries. Clinical Microbiology Reviews, 34(2), e00087-19. 33627442 10.1128/CMR.00087-19 PMC8549823

[35] Zahedi A, Ryan U. 2020. *Cryptosporidium* – An update with an emphasis on foodborne and waterborne transmission. Research in Veterinary Science, 132, 500–512. 32805698 10.1016/j.rvsc.2020.08.002

[36] Zhang K, Fu Y, Li J, Zhang L. 2021. Public health and ecological significance of rodents in *Cryptosporidium* infections. One Health, 14, 100364. 34984218 10.1016/j.onehlt.2021.100364 PMC8692995

[37] Zhao W, Ren G, Jiang W, Wang L, Wang J, Yuan Z, Yan L, Li Y, Sun Y, Xue X, Jiang Y, Lu G, Huang H. 2024. Genetic characterizations of *Cryptosporidium* spp. from children with or without diarrhea in Wenzhou, China: High probability of zoonotic transmission. BMC Microbiology, 24(1), 113. 38575881 10.1186/s12866-024-03273-w PMC10993503

[38] Zhao W, Zhou H, Huang Y, Xu L, Rao L, Wang S, Wang W, Yi Y, Zhou X, Wu Y, Ma T, Wang G, Hu X, Peng R, Yin F, Lu G. 2019. *Cryptosporidium* spp. in wild rats (*Rattus* spp.) from the Hainan Province, China: Molecular detection, species/genotype identification and implications for public health. International Journal for Parasitology: Parasites and Wildlife, 9, 317–321. 31338292 10.1016/j.ijppaw.2019.03.017 PMC6626849

[39] Zhao W, Wang J, Ren G, Yang Z, Yang F, Zhang W, Xu Y, Liu A, Ling H. 2018. Molecular characterizations of *Cryptosporidium* spp. and *Enterocytozoon bieneusi* in brown rats (*Rattus norvegicus*) from Heilongjiang Province, China. Parasites & Vectors, 11(1), 313. 29793513 10.1186/s13071-018-2892-7 PMC5968579

[40] Zou Y, Ma JG, Yue DM, Zheng WB, Zhang XX, Zhao Q, Zhu XQ. 2017. Prevalence and risk factors of *Cryptosporidium* infection in farmed pigs in Zhejiang, Guangdong, and Yunnan provinces, China. Tropical Animal Health and Production, 49(3), 653–657. 28168399 10.1007/s11250-017-1230-y

